# Sleep quality, sleep duration, and sleep disturbances among hospital night workers: a prospective cohort study

**DOI:** 10.1007/s00420-023-02033-z

**Published:** 2023-12-28

**Authors:** Fleur van Elk, Bette Loef, Karin I. Proper, Alex Burdorf, Suzan J. W. Robroek, Karen M. Oude Hengel

**Affiliations:** 1 Department of Public Health, Erasmus University Medical Center, P.O. Box 2040, 3000 CA Rotterdam, The Netherlands; 2 Center for Nutrition, Prevention and Health Services, National Institute for Public Health and the Environment, Bilthoven, The Netherlands; 3 Department of Public and Occupational Health, Amsterdam Public Health Research Institute, Amsterdam UMC, Vrije Universiteit Amsterdam, Amsterdam, The Netherlands; 4 Department of Work Health Technology, Netherlands Organisation for Applied Scientific Research TNO, Leiden, The Netherlands

**Keywords:** Night workers, Healthcare, Sleep components, Between–within Poisson regression

## Abstract

**Purpose:**

This study aimed to assess among hospital night workers (i) to what extent sleep quality, sleep duration and sleep disturbances overlap, and (ii) associations between sociodemographic factors, lifestyle factors and work characteristics and sleep components.

**Methods:**

Data were used from 467 hospital night workers participating in the Klokwerk + study, a prospective cohort study with two measurements. Sleep quality was measured by the Pittsburgh Sleep Quality Index, sleep duration and sleep disturbances were measured by the Medical Outcomes Study Sleep Scale. The overlap between the three sleep measures was visualized with a Venn diagram and the proportions of overlap was calculated. Associations between independent variables (sociodemographic factors, lifestyle factors and work characteristics) and the three sleep outcomes were estimated using between–within Poisson regression models.

**Results:**

About 50% of the hospital night workers had at least one poor sleep outcome. Overlap in poor sleep outcomes was apparent for 36.8% of these workers, while the majority had a poor outcome in one of the sleep components only (63.1%). Former smoking had a significant association with poor sleep quality. For most independent variables no associations with poor sleep outcomes were observed.

**Conclusion:**

Our findings suggest that sleep quality, sleep duration and sleep disturbances are separate entities and should be studied separately. Lifestyle factors and work characteristics were generally not associated with poor sleep. Since these factors can have an acute effect on sleep, future research should consider ecological momentary assessment to examine how exposure and outcomes (co)vary within-persons, over time, and across contexts.

*Trial registration* Netherlands Trial Register trial number NL56022.041.16.

**Supplementary Information:**

The online version contains supplementary material available at 10.1007/s00420-023-02033-z.

## Introduction

Night work can negatively affect health of workers, mostly due to disturbances in the circadian rhythm and a lack of sleep (Arendt 2010; Rosa et al. 2019). Night workers have a longer sleep-onset latency and total sleep duration, and wake up more often during sleep (Chang and Peng 2021b). Healthcare workers are a specific group of night workers whose presence at work is essential 24 h a day. Night workers in healthcare experience worse sleep quality (Chang and Li 2022; Chang and Peng 2021a; McDowall et al. 2017), and more often a shorter or longer sleep period than non-night workers in healthcare (Chang and Li 2022; Hulsegge et al. 2019).

Many studies that conduct research on sleep use different components of sleep. It needs to be considered that these different components such as sleep quality and sleep duration are different fundamental components of sleep. Differences between factors that are related to sleep quality and sleep duration (such as health, fatigue, and sleepiness) have indeed already been suggested (Kohyama 2021; Pilcher et al. 1997). In addition, sleep disturbances are considered an important aspect of sleep, whereas it is usually used as part of measuring sleep quality (Yi et al. 2006). Therefore, it is of interest to what extent sleep quality, sleep duration, and sleep disturbances overlap.

Several studies have reported associations for sociodemographic factors, lifestyle factors, and work characteristics with different sleep components. For example, ageing is related to more sleep disturbances in night workers (Härmä et al. 1994; Pires et al. 2009), and night-working women tend to experience worse sleep quality than night-working men (Park and Suh 2020). Although studies on the association between lifestyle factors and sleep outcomes among night workers are lacking, meta-analyses in the general population show associations between lifestyle factors and sleep. In these meta-analyses, high physical activity was associated with reduced sleep duration the following night at the inter-individual level (Atoui et al. 2021), smoking was associated with a number of sleep-related issues (Amiri and Behnezhad 2020), and general alcohol consumption with the onset of sleep disorders (Hu et al. 2020). Screen use is recently receiving more attention as lifestyle factor that could influence sleep due to exposure to light. A brief review among adults and children showed that more screen time was associated with lower quality and duration of sleep, and a higher likelihood of later bedtimes and difficulties staying asleep (Hale et al. 2015). Furthermore, several work-related factors were also associated with poor sleep. A meta-analysis showed that job demands such as hours worked and workload were negatively associated with sleep quality and sleep duration, while job support was positively associated with sleep quality and sleep duration (Litwiller et al. 2017). To date, most of this research has focused on general working populations and studies among night workers are mainly lacking. Since night work is common in healthcare to ensure continuity in care, and because of disturbed sleep among these night workers, more research is needed among this group. In addition, this research should not only focus on the effects of working nights in general, but also on other risk factors concerning lifestyle and work characteristics that might influence sleep. Moreover, it is important to study the between and within associations of lifestyle and work-related factors, since it is known that both behaviors such as physical activity and alcohol consumption not only differs between individuals but can also differ within an individual over time (e.g., sleep can differ due to seasonal and weather effects (Mattingly et al. 2021)) The specific insights of the different associations with sleep among night workers are necessary to design effective interventions to improve the sleep of night workers with poor sleep outcomes.

Against this background, this study aims to assess (i) to what extent sleep quality, sleep duration, and sleep disturbances overlap among hospital workers with night shifts, and (ii) which sociodemographic factors, lifestyle factors, and work characteristics are associated with sleep quality, sleep duration, and sleep disturbances among hospital workers with night shifts.

## Methods

### Data and study population

Data from the Klokwerk + study, which is a prospective cohort study, were used (Loef et al. 2016). The aim of Klokwerk + was to study the effects of shift work on infection susceptibility and body weight, and the mechanisms underlying the health effects of shift work. The study population consisted of 611 healthcare workers aged 18–65 years from six different hospitals in the Netherlands. Hospital workers were nurses, physicians, and other (allied) health professionals. The first measurement (T0) consisting of a questionnaire and anthropometric measurements took place in September–December 2016 and the second measurement (T1) after 6 months in April–June 2017. For the current study, only hospital workers with night shifts (*n* = 467 at T0 and *n* = 388 at T1) were included. Working night shifts was defined as working shifts between 00:00 and 06.00 AM at the time of the study period.

The institutional review board of the University Medical Center Utrecht in Utrecht, The Netherlands approved the current study on March 15, 2016 (study protocol number 16–044/D, NL56022.041.16). Written informed consent was obtained from all participants.

### Measures

#### Outcome measures

*Sleep quality.* Sleep quality was measured using a single item of the Pittsburgh Sleep Quality Index (PSQI) which asks participants to indicate how they rate their overall sleep quality in the past month on a 4-point Likert scale (ranging from very good to very bad) (Buysse et al. 1989). Poor sleep quality was defined as having a very or fairly bad sleep quality.

*Sleep duration.* Sleep duration was derived from the Medical Outcomes Study (MOS) Sleep Scale, which measures the general usual sleep habits in the past 4 weeks (Hays et al. 2005). The MOS consists of 12 items covering 6 dimensions. One of the items measures duration of sleep by asking to report the amount of hours of sleep per day. Based on the amount of hours slept per day, the measure of sleep duration was dichotomized into recommended sleep duration (7–9 h per day) and non-recommended sleep duration (< 7 or ≥ 9 h per day) (Hirshkowitz et al. 2015).

*Sleep disturbances.* Sleep disturbances is one of the dimensions of the MOS Sleep Scale, and is based on four items that are scored on a 6-point Likert scale (Hays et al. 2005). The items concern problems falling asleep, how long it takes to fall asleep, not having a quiet sleep, and having problems falling asleep again after waking up during sleep time. The crude scores on the 6-point Likert scale were transformed to a 0–100% range. The percentages of the four items were averaged together into a 0–100 continuous dimension score, and a higher percentage indicated more sleep disturbances. Because of a skewed distribution, the dimension score was then dichotomized so that the upper quartile of sleep disturbances would be compared to the rest. The outcome measures were measured at both T0 and T1.

#### Sociodemographic factors

Age, sex, partner status, level of education, occupation, and chronotype were included as sociodemographic factors. These factors were only measured at T0, except for occupation. Age was a continuous variable, and sex was a dichotomous variable (male/female). Partner status was dichotomized into living together with a partner and not living together with a partner. Level of education was dichotomized into lower (elementary school to vocational education) and higher (higher vocational education/university) educated. Occupation was dichotomized into nurse and other (e.g., physicians, paramedics, caregiver). Self-reported chronotype was divided into three categories based on a self-rated single item of the Munich ChronoType Questionnaire (MCTQ): morning type, evening type, and no specific type (Roenneberg et al. 2003).

#### Lifestyle factors

Body Mass Index (BMI), physical activity, smoking, alcohol use, and screen use were included as lifestyle factors and were measured at both T0 and T1. BMI was calculated at baseline and follow-up based on weight and height measurements performed by the research team and divided into three categories: normal weight including underweight (BMI < 25 kg/m^2^), overweight (BMI 25–30 kg/m^2^), and obesity (BMI ≥ 30 kg/m^2^) (WHO 2018). Physical activity was measured with the Short Questionnaire to ASsess Health enhancing physical activity (SQUASH) (Wendel-Vos et al. 2003). The number of hours per week was calculated for three categories: sports activities, activity at work, and other activities (including commuting between work and home, leisure time activity excluding sports, and domestic chores). Smoking status was divided into three categories: never smoker, former smoker, and current smoker. Alcohol use was dichotomized into > 7  glasses per week and ≤ 7  glasses per week according to the recommended intake by the Dutch Health Council (Gezondheidsraad 2015). Screen use was based on a self-constructed questionnaire about the amount of times a week devices using lights (such as television, computer, smartphone, tablet) were used in the hour before sleep. Screen use was dichotomized into less than every day of the week and every day of the week.

#### Work characteristics

Working hours, amount of years working in night shifts, and average amount of night shifts per month were included as work characteristics and were measured at both T0 and T1. Working hours was divided into three categories: ≤ 24 h per week, 25–35 h per week and ≥ 36 h per week (fulltime). Amount of years working in night shifts was categorized into < 10 years, 10–19 years, and ≥ 20 years, as was done previously (Loef et al. 2019). The average amount of night shifts per month was categorized into 1–2 per month, 3–4 per month, and ≥ 5 per month (Loef et al. 2019).

### Statistical analysis

Lifestyle factors and working characteristics could change between the two measurements over time (T0 and T1). Therefore, an analysis of the variance (ANOVA) was performed to explore how much variance of time-varying dependent and independent variables were attributed to between- and to within-individuals variation. Given the presence of within-individual variation between T0 and T1, between–within Poisson regression models were fitted to investigate associations between independent variables and three sleep outcomes, while taking into account within-individual variation between T0 and T1. In these models, between-individuals estimates were derived by including the person-specific overall mean of the time-varying variables, and within-individuals estimates by including the deviations from the person-specific mean of the time-varying variables. The focus of the current study was on the differences in associations between individuals, while taking into account the within-individuals variations. Poisson regression models were chosen rather than logistic regression analyses because of the dichotomous outcomes with a high prevalence. Dichotomous outcomes were chosen because of a skewed distribution of the variables.

First, the overlap between poor sleep quality, non-recommended sleep duration, and more sleep disturbances in the study population was visualized with a Venn diagram. Weighted Cohen’s *κ* were calculated to determine the agreement between poor sleep outcomes, based on the proportion of agreement over and above chance agreement. A weighted Cohen’s *κ* of less than 0 shows no agreement, 0–0.20 shows slight, 0.21–0.40 fair, 0.41–0.60 moderate, 0.61–0.80 substantial, and 0.81–1.0 perfect agreement (Landis and Koch 1977).

Second, for each of the three sleep outcomes, crude univariate analyses were performed with the sleep outcomes as dependent variables and sociodemographic factors, lifestyle factors, and work characteristics as independent variables. Then, for each of the sleep outcome, multivariable analyses were performed including all sociodemographic factors, lifestyle factors, and work characteristics in the model.

All analyses were performed using IBM SPSS Software version 28.

## Results

Table 1 shows the baseline characteristics of the hospital night workers. The majority was female (86.9%) and nurse (83.3%), and with a mean age of 40 years. More than half of the workers were higher educated (55.0%), and over 40% had an evening chronotype. The largest percentages of night workers worked less than 10 years (37.3%) or 20 or more years (38.1%) in night shifts, and 3–4 (42.8%) or 5 or more (44.3%) night shifts per month. The minority experienced very/fairly poor sleep quality (18.4%), non-recommended sleep duration (29.3%), and more sleep disturbances (28.1%). The between-individual variance in all sleep components, sociodemographic factors, lifestyle factors, and work characteristics was much larger than the within-individual variance (Supplementary Table 1).

**Table 1:** Characteristics of hospital workers with night shifts (*n* = 467) at baseline

	Baseline (*n* = 467)
	% or mean (SD)
Age (in years)	40.28 (12.06)
Female	406 (86.9%)
Living together	344 (73.7%)
Lower educated	210 (45.0%)
Nurse	389 (83.3%)
Chronotype	
Morning type	162 (34.7%)
Evening type	194 (41.5%)
No specific type	109 (23.3%)
Body mass index	
Normal weight	248 (53.1%)
Overweight	156 (33.4%)
Obese	63 (13.5%)
Physical activity (in hours/week)	
Sports	2.75 (3.27)
Occupational physical activity	24.34 (11.83)
Other physical activity	24.41 (16.29)
Smoking	
Never smoker	295 (63.2%)
Former smoker	115 (24.6%)
Current smoker	55 (11.8%)
Alcohol use	
0 glasses per week	178 (38.1%)
1–7 glasses per week	186 (39.8%)
> 7 glasses per week	101 (21.6%)
Screen use 1 h before sleep less than every day of the week	144 (30.8%)
Working hours	
≤ 24 h per week	82 (17.6%)
25–35 h per week	226 (48.4%)
≥ 36 h per week (fulltime)	159 (34.0%)
Years with night work	
< 10 years	174 (37.3%)
10–19 years	114 (24.4%)
≥ 20 years	178 (38.1%)
Night shifts per month	
1–2 per month	60 (12.8%)
3–4 per month	200 (42.8%)
≥ 5 per month	207 (44.3%)
Very/fairly poor sleep quality	86 (18.4%)
Non-recommended sleep duration (< 7 or ≥ 9 h per day)	137 (29.3%)
More sleep disturbances (upper quartile)	131 (28.1%)

*SD* standard deviation

**Fig. 1:**
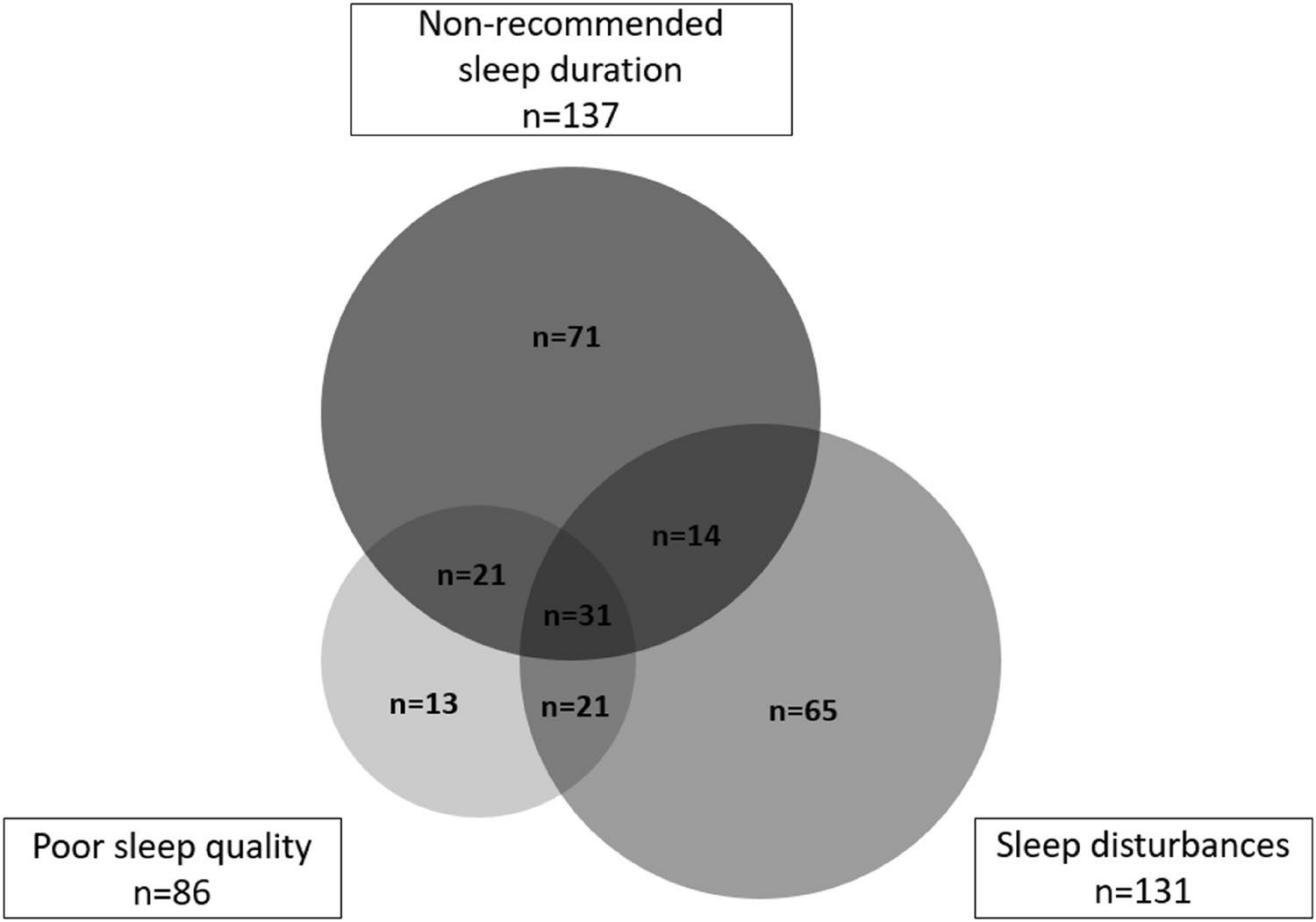
Venn diagram of 236 individuals experiencing poor sleep quality, non-recommended sleep duration, and more sleep disturbances

In total, half of the night workers (50.5%, *n* = 236) had a poor outcome in at least one sleep component at baseline. Among these persons with a poor sleep outcome 63.1% had one poor outcome, 23.7% had two poor outcomes, and 13.1% had three poor outcomes (Fig. 1). Table 2 shows a higher prevalence of poor sleep quality among persons with non-recommended sleep duration (38.0%) or more sleep disturbances (39.7%) compared to the prevalence in the total study population (18.4%). In particular among those with poor sleep quality the prevalence of non-recommended sleep duration and more sleep disturbances is high. Poor sleep quality had a fair agreement with non-recommended sleep duration (*κ* = 0.31, 95% CI 0.21, 0.40) and with more sleep disturbances (*κ* = 0.33, 95% CI 0.23, 0.43). Slight agreement was found between non-recommended sleep duration and more sleep disturbances (*κ* = 0.07, 95% CI − 0.03, 0.16).

**Table 2:** Co-occurrence of poor sleep outcomes based on conditional proportions of poor sleep outcomes among hospital workers with night shifts at baseline (*n* = 467)

Sleep outcome	Prevalence		No poor outcome in other sleep components, conditional on prevalence of sleep outcome		Concurrent poor sleep outcomes (%) Conditional proportions		
	*n*	%	*n*	%	Poor sleep quality	Non-recommended sleep duration	Sleep disturbances
Poor sleep quality	86	18.4	13	15.1	–	60.5	60.5
Non-recommended sleep duration	137	29.3	71	51.8	38.0	–	32.8
Sleep disturbances	131	28.1	65	49.6	39.7	34.4	–

Weighted Cohen’s *κ* (95% Confidence Interval): poor sleep quality and non-recommended sleep duration 0.31 (0.25, 0.40), poor sleep quality and sleep disturbances 0.33 (0.23, 0.43), non-recommended sleep duration and sleep disturbances 0.07 (− 0.03, 0.16)

The multivariable models (Table 3) based on between–within analysis showed a statistically significant association between former smoking and poor sleep quality only (RR 1.71, 95% CI 1.09, 2.67). Concerning sociodemographic factors, some modest and non-statistically significant associations were found. Individuals not living together with a partner were 1.20–1.40 times more likely to have poor sleep outcomes, and morning and evening types were respectively 1.32 and 1.48 times more likely to have more sleep disturbances than individuals with no specific chronotype. Concerning lifestyle factors and work characteristics, again some modest and non-statistically significant associations were found with the sleep components. Supplementary Tables 2 and 3 show the crude univariate models for respectively the between-individuals and within-individuals comparisons.

**Table 3:** Associations of sociodemographic factors, lifestyle factors, and work characteristics with sleep quality, sleep duration, and sleep disturbances among 467 hospital workers, estimated with multivariable between–within Poisson regression analysis

	Poor sleep quality	Non-recommended sleep duration	Sleep disturbances
	RR (95% CI)	RR (95% CI)	RR (95% CI)
Age (in years)^a^	1.00 (0.97, 1.03)	1.02 (0.99, 1.04)	0.99 (0.96, 1.01)
Female^a^	1.14 (0.60, 2.18)	0.77 (0.48, 1.22)	1.40 (0.78, 2.52)
Not living together^a^	1.38 (0.90, 2.12)	1.20 (0.85, 1.70)	1.40 (0.99, 2.00)
Higher educated^a^	0.93 (0.63, 1.38)	1.12 (0.82, 1.52)	0.76 (0.55, 1.04)
Nurse (ref. other occupation)	0.74 (0.44, 1.26)	1.01 (0.66, 1.54)	0.86 (0.56, 1.34)
Chronotype (ref. no specific type)^a^			
Morning type	1.17 (0.70, 1.94)	1.08 (0.75, 1.56)	1.32 (0.87, 2.00)
Evening type	1.26 (0.78, 2.05)	0.88 (0.61, 1.28)	1.48 (0.99, 2.21)
Body mass index (ref. normal weight)			
Overweight	0.85 (0.55, 1.32)	0.87 (0.62, 1.22)	1.17 (0.84, 1.64)
Obese	1.27 (0.74, 2.18)	1.40 (0.92, 2.14)	1.06 (0.66, 1.69)
More physical activity (in hours/week)			
Sports	0.94 (0.86, 1.01)	0.98 (0.93, 1.03)	0.97 (0.91, 1.03)
Activity at work	0.99 (0.97, 1.01)	0.99 (0.97, 1.01)	0.99 (0.98, 1.01)
Other activity	1.01 (1.00, 1.03)	1.01 (1.00, 1.02)	1.00 (0.99, 1.01)
Smoking (ref. never smoker)			
Former smoker	**1.71 (1.09, 2.67)**	1.41 (0.99, 2.01)	1.18 (0.81, 1.72)
Current smoker	1.08 (0.56, 2.09)	1.06 (0.63, 1.78)	1.06 (0.64, 1.77)
Glasses of alcohol per week (ref. 0 glasses)			
1–7 glasses per week	0.81 (0.51, 1.27)	0.85 (0.59, 1.21)	0.79 (0.54, 1.14)
> 7 glasses per week	0.95 (0.56, 1.59)	0.86 (0.56, 1.30)	1.18 (0.79, 1.78)
Daily screen use 1 h before sleep	0.96 (0.60, 1.54)	1.08 (0.74, 1.58)	1.24 (0.85, 1.81)
Working hours (ref. ≤ 24 h per week)			
25–35 h per week	1.24 (0.69, 2.23)	1.38 (0.85, 2.23)	1.14 (0.73, 1.79)
≥ 36 h per week	1.62 (0.80, 3.27)	1.65 (0.94, 2.88)	1.29 (0.73, 2.28)
Years with night work (ref. < 10 years)			
10–19 years	0.93 (0.51, 1.69)	0.73 (0.46, 1.16)	1.22 (0.75, 1.99)
≥ 20 years	0.90 (0.41, 1.96)	0.57 (0.32, 1.03)	1.64 (0.88, 3.05)
Night shifts per month (ref. 1–2 per month)			
3–4 per month	1.05 (0.52, 2.11)	1.10 (0.62, 1.95)	0.80 (0.48, 1.33)
≥ 5 per month	1.06 (0.52, 2.18)	1.24 (0.70, 2.17)	0.59 (0.34, 1.01)

Numbers depicted in bold are statistically significant

Sleep quality: 0 = good, 1 = bad. Sleep duration: 0 = recommended sleep duration (7–9 h per day), 1 = non-recommended sleep duration (< 7 of ≥ 9 h per day). Sleep disturbances: 0 = less disturbances (lowest 75% sleep disturbances), 1 = more disturbances (highest 25% sleep disturbances)

*RR* relative risk, 95% *CI* 95% confidence interval

^a^Only measured at T0, making them time independent factors

## Discussion

Half of the night workers in the current study had a poor sleep quality, non-recommended sleep duration, or sleep disturbances. Of these individuals, almost two-thirds reported one poor sleep outcome only, with non-recommended sleep duration being reported most often. The relatively high number of night workers that reported one specific poor sleep outcome indicates that the components of sleep are partly separate entities rather than completely interchangeable. By disentangling sleep duration and sleep disturbances from subjective sleep quality, we were able to show that an overarching assessment of sleep quality is only one aspect of the multidimensional concept of sleep. Except for former smoking, no significant associations of sociodemographic factors as well as lifestyle factors and work characteristics with poor sleep outcomes were found between individuals while taking into account the within-individuals comparisons.

Sleep is necessary for health and wellbeing, which is shown by the relation of sleep problems such as insomnia with health and function problems (Buysse 2013). Our research showed that components of sleep differ from each other. Night workers with poor sleep quality were also more likely to have non-recommended sleep duration and sleep disturbances. It is possible that subjective sleep quality is a more overarching component which partly depends on sleep duration and sleep disturbances. However, limited overlap existed between non-recommended sleep duration and sleep disturbances. In addition, also among persons with non-recommended sleep duration and sleep disturbances less than 40% experienced poor sleep quality. This indicates that non-recommended sleep duration and sleep disturbances can occur without subjective poor sleep quality. This corroborates a study among the general population showing differences between health factors related to sleep quality and sleep duration (Kohyama 2021). Many studies have assessed sleep quality by combining the prevalence of a certain set of sleep disturbances, for example with the widely used Pittsburgh Sleep Quality Index (PSQI) (Buysse et al. 1989). Because of partly separate entities of sleep components, future research on sleep needs to differentiate between sleep quality and sleep disturbances. The lack of overlap between non-recommended sleep duration and sleep disturbances was also revealed in studies focusing on the influence of poor sleep on health outcomes. For example, overweight in young adults and youth was associated with sleep disturbances rather than sleep duration (Jarrin et al. 2013; Vargas et al. 2014). Likewise, sleep disturbances and long sleep duration, rather than short sleep duration, were associated with inflammatory diseases (Irwin et al. 2016), and poorer sleep quality was related to poorer physical quality of life, while short sleep duration was not (Matsui et al. 2021). To further clarify the differences between sleep quality, sleep duration and sleep disturbances, we recommend future research on sleep questionnaires that can better distinguish between the different sleep components. In addition, to further concretize the sleep components, objective sleep measures using motion watches could be useful.

Regarding sociodemographic characteristics, no significant associations were found with any of the sleep outcomes. For lifestyle factors, the only surprising finding was the relation between smoking and poor sleep outcomes, which was stronger for former smokers than for current smokers. It is difficult to explain this incidental finding. It could be hypothesized that sleep problems may have prompted individuals to quit smoking. A possible reason for the lack of associations between lifestyle factors and sleep disturbance is the relatively healthy study population, which might be explained by the healthy worker effect, meaning that healthcare workers who are able to cope with working night shifts stayed and others stopped working in night shifts. For work characteristics also, only non-statistically significant associations were found. In contrast, previous research showed evidence for a positive relation between night shift-related schedule characteristics, such as rotation, quick returns and number of consecutive night shifts, and poor sleep outcomes (e.g., Van de Ven et al. 2021). Our results might differ from these results due to the fact that effects of work characteristics such as number of consecutive night shifts on sleep are most likely acute effects, which are present directly after consecutive night shifts (Van de Ven et al. 2021), whereas our study focused on sleep outcomes in the past month. As sleep of healthcare workers in night work vary day-by-day, it is important to gain insight in this variation in sleep over time, but also in what features of their working lives predicts such variability. Due to current mobile technologies, an ecological momentary assessment (EMA) provides the opportunity to examine how exposure and outcomes vary and co-vary within-persons, over time, and across contexts. EMA could also be interesting to study acute effects of lifestyle behaviors on sleep. The advantages of EMA are that data are collected in real-world settings (ecological) focusing on the current state of the participant (momentary), reducing recall bias (Shiffman et al. 2008). Therefore, acute effects of lifestyle behaviors and work characteristics on sleep can be measured, whereby it is possible to combine both subjective and objective measures. In addition, the current study focused on job characteristics rather than work-related factors, whereas meta-analysis showed that psychosocial risk factors (e.g., demands and support) were related to sleep quality and sleep duration (Litwiller et al. 2017). This concerned the general working population, whereas it would be interesting to study the relation between psychosocial risk factors and sleep among night workers in healthcare who are already at risk for poor sleep outcomes. Therefore, it is highly recommended to include psychosocial work-related factors in future research on the association between work and sleep among night workers.

### Strengths and limitations

One of the strengths of the current study is the possibility to distinguish between different components of sleep in order to study the overlap of those components. Another strength are the two measurements over time, making it possible to also take into account within-individual effects, which is especially important for factors that change over time.

However, some limitations also need to be considered. First, the data on sleep components were based on self-report. It would be relevant to study the relation between different factors and objectively measured sleep outcomes with large numbers of participants. Second, the dichotomization of the sleep disturbances outcome is arbitrary because the cut-off point was based on the distribution of the data, whereas the raw scores indicate that we reached the hospital workers with night shifts with relatively limited sleep disturbances. Third, whereas we were able to take into account the within-individual effects when studying the between-individual effects, it was not feasible to focus on within-individuals comparisons because the short time interval between the two measurements limits to study differences within individuals for factors that could have changed over a longer period of time (e.g., BMI, physical activity), and other factors that were studied are more persistent and unlikely to vary much over time (e.g., smoking, working hours, number of night shifts). More research is needed to study the associations over multiple measurement periods and to get better insights into within-individuals differences. A possibility is to adopt EMA designs.

## Conclusion

Our findings suggest that sleep quality, sleep duration and sleep disturbances are partly separate entities and therefore need to be studied separately. Future research on the effects of work on sleep among night workers may consider EMA designs and include lifestyle factors as well as work-related factors such as psychosocial job demands and resources to study acute effects on sleep outcomes.

### Supplementary Information

Below is the link to the electronic supplementary material.

Supplementary file1 (PDF 617 kb)

## Data Availability

Data are stored at RIVM, Center for Nutrition, Prevention and Health Services in The Netherlands. Data are available upon reasonable request by the second author.
