# Supplementary material for: Sleep quality, sleep duration, and sleep disturbances among hospital night workers: a prospective cohort study
Source: Int Arch Occup Environ Health. 2023 Dec 28;97(2):179–88. doi: 10.1007/s00420-023-02033-z (PMC10876714; doi:10.1007/s00420-023-02033-z)
Supplement: Supplementary file 1 — Supplementary file1 (PDF 617 kb) [file 420_2023_2033_MOESM1_ESM.pdf]

Sleep quality, sleep duration, and sleep disturbances among hospital night workers: a prospective cohort study

International Archives of Occupational and Environmental Health

- Fleur van Elk, MSc – Department of Public Health, Erasmus University Medical Center, Rotterdam, The Netherlands, f.vanelk@erasmusmc.nl
- Bette Loef, PhD – Center for Nutrition, Prevention and Health Services, National Institute for Public Health and the Environment, Bilthoven, The Netherlands, bette.loef@rivm.nl
- Karin I. Proper, Prof. Dr. – Center for Nutrition, Prevention and Health Services, National Institute for Public Health and the Environment, Bilthoven, The Netherlands, karin.proper@rivm.nl & Department of Public and Occupational Health, Amsterdam UMC, Vrije Universiteit Amsterdam, Amsterdam Public Health research institute, Amsterdam, The Netherlands
- Alex Burdorf, Prof. Dr. – Department of Public Health, Erasmus University Medical Center, Rotterdam, The Netherlands, a.burdorf@erasmusmc.nl
- Karen M. Oude Hengel, PhD – Department of Work Health Technology, Netherlands Organisation for Applied Scientific Research TNO, Leiden, The Netherlands, karen.oudehengel@tno.nl & Department of Public Health, Erasmus University Medical Center, Rotterdam, The Netherlands, k.oudehengel@erasmusmc.nl
- Suzan J.W. Robroek, PhD – Department of Public Health, Erasmus University Medical Center, Rotterdam, The Netherlands, s.robroek@erasmusmc.nl

Corresponding author: Karen M. Oude Hengel, Erasmus University Medical Center, Department of Public Health, P.O. Box 2040, 3000 CA Rotterdam, The Netherlands, k.oudehengel@erasmusmc.nl

Supplementary table 1. Variation in sleep factors and time-dependent lifestyle factors, and work characteristics between baseline and 6 months follow-up between individuals and within individuals among 467 hospital workers with night shifts.

	Score		Variance	
	Baseline mean (SD*)	Follow-up** mean (SD*)	Between individuals %	Within individuals %
**Sleep factors**				
Sleep quality	0.84 (0.37)	0.82 (0.39)	69.9	30.1
Sleep duration	0.72 (0.45)	0.72 (0.45)	71.2	28.8
Sleep disturbances	0.73 (0.44)	0.73 (0.45)	76.2	23.6
**Socio-demographic factors**				
Occupation	0.83 (0.37)	0.83 (0.38)	97.4	2.6
**Lifestyle factors**				
Body Mass Index				
Normal weight	0.53 (0.50)	0.54 (0.50)	97.3	2.7
Overweight	0.33 (0.47)	0.33 (0.47)	91.7	8.3
Obesity	0.13 (0.34)	0.12 (0.33)	89.0	11.0
Physical activity (h/week):				
Sports	2.75 (3.28)	2.73 (2.81)	79.4	20.6
Occupational physical activity	24.30 (11.69)	24.03 (11.69)	67.7	32.3
Other physical activity	24.37 (15.50)	24.02 (14.81)	79.1	20.9
Smoking				
Never	0.66 (0.48)	0.66 (0.47)	95.1	4.8
Former	0.23 (0.42)	0.24 (0.43)	93.0	7.1
Current	0.11 (0.32)	0.10 (0.30)	91.3	8.7
Alcohol use:				
0 glasses per week	0.38 (0.49)	0.40 (0.49)	89.1	10.9
1-7 glasses per week	0.40 (0.49)	0.42 (0.49)	78.6	21.4
>7 glasses per week	0.22 (0.41)	0.18 (0.39)	83.9	16.1
Screen use	0.68 (0.47)	0.67 (0.47)	76.8	23.0
**Work characteristics**				
Working hours:				
≤24 hours per week	0.18 (0.38)	0.19 (0.39)	89.6	10.4
25-35 hours per week	0.48 (0.50)	0.49 (0.50)	87.0	13.0
≥36 hours per week	0.34 (0.47)	0.32 (0.47)	90.5	9.5
Years with night work:				
<10 years	0.37 (0.48)	0.33 (0.47)	94.7	5.3
10-19 years	0.24 (0.43)	0.26 (0.44)	88.6	11.4
≥20 years	0.38 (0.49)	0.41 (0.49)	95.3	4.7
Night shifts per month:				
1-2 per month	0.13 (0.33)	0.14 (0.35)	80.1	19.9
3-4 per month	0.43 (0.50)	0.46 (0.50)	72.0	28.0
≥5 per month	0.44 (0.50)	0.39 (0.49)	78.6	21.4

*SD = standard deviation.

**Follow-up was 6 months after the baseline measurement.

Supplementary table 2. Crude between-individual associations of sociodemographic factors, lifestyle factors, and work characteristics with sleep quality, sleep duration, and sleep disturbances among 467 hospital workers, estimated with univariate between-within Poisson regression analyses.

	Poor sleep quality	Non-recommended sleep duration	Sleep disturbances
	RR (95% CI)**	RR (95% CI)**	RR (95% CI)**
**Age (in years)***	1.00 (0.99, 1.02)	1.01 (1.00, 1.02)	1.00 (0.99, 1.01)
**Female***	1.04 (0.65, 1.69)	0.73 (0.52, 1.02)	**1.64 (1.03, 2.62)**
**Not living together***	1.39 (0.99, 1.94)	1.22 (0.93, 1.61)	**1.36 (1.03, 1.79)**
**Higher educated***	0.91 (0.66, 1.24)	1.09 (0.85, 1.41)	**0.74 (0.57, 0.95)**
**Nurse (ref. other occupation)**	0.88 (0.57, 1.36)	0.94 (0.67, 1.33)	1.06 (0.73, 1.52)
**Chronotype (ref. no specific type)***			
Morning type	1.27 (0.81, 1.97)	1.03 (0.74, 1.44)	1.42 (0.97, 2.07)
Evening type	1.39 (0.91, 2.12)	0.98 (0.71, 1.36)	**1.63 (1.14, 2.34)**
**Body Mass Index (ref. normal weight)**			
Overweight	1.09 (0.75, 1.59)	0.96 (0.71, 1.30)	1.19 (0.88, 1.60)
Obese	**1.72 (1.11, 2.69)**	**1.50 (1.05, 2.16)**	1.26 (0.84, 1.88)
**More physical activity (in hours/week)**			
Sports	**0.93 (0.86, 1.00)**	0.99 (0.94, 1.04)	0.95 (0.90, 1.00)
Occupational physical activity	0.99 (0.98, 1.01)	1.00 (0.99, 1.01)	0.99 (0.98, 1.01)
Other physical activity	1.00 (0.99, 1.02)	1.00 (0.99, 1.01)	1.00 (0.99, 1.01)
**Smoking (ref. never smoker)**			
Former smoker	**1.70 (1.19, 2.42)**	**1.44 (1.08, 1.93)**	1.30 (0.96, 1.75)
Current smoker	1.34 (0.80, 2.25)	1.24 (0.82, 1.87)	1.24 (0.82, 1.88)
**Glasses of alcohol per week (ref. 0 glasses)**			
1-7 glasses per week	0.85 (0.58, 1.25)	0.83 (0.61, 1.14)	0.79 (0.57, 1.09)
>7 glasses per week	1.07 (0.69, 1.65)	1.04 (0.74, 1.48)	1.15 (0.81, 1.63)
**Daily screen use 1 hour before sleep**	1.10 (0.74, 1.62)	1.25 (0.91, 1.71)	1.20 (0.87, 1.65)
**Working hours (ref. ≤24 hours per week)**			
25-35 hours per week	1.24 (0.76, 2.02)	1.40 (0.92, 2.13)	1.11 (0.76, 1.61)
≥36 hours per week	1.25 (0.75, 2.07)	**1.69 (1.11, 2.57)**	0.95 (0.64, 1.42)
**Years with night work (ref. <10 years)**			
10-19 years	1.03 (0.67, 1.59)	0.97 (0.69, 1.36)	1.00 (0.70, 1.43)
≥20 years	1.00 (0.69, 1.45)	0.95 (0.71, 1.27)	1.13 (0.84, 1.52)
**Night shifts per month (ref. 1-2 per month)**			
3-4 per month	1.06 (0.58, 1.93)	1.13 (0.68, 1.82)	0.91 (0.58, 1.41)
≥5 per month	1.38 (0.78, 2.42)	1.52 (0.96, 2.42)	0.86 (0.56, 1.32)

Numbers depicted in bold are statistically significant.

Sleep quality: 0 = good, 1 = bad. Sleep duration: 0 = recommended sleep duration (7-9 hours per day), 1 = non-recommended sleep duration (<7 of ≥ 9 hours per day). Sleep disturbances: 0 = less disturbances (lowest 75% sleep disturbances), 1 = more disturbances (highest 25% sleep disturbances).

*Only measured at T0, making them time independent factors.

**RR = relative risk, 95% CI = 95% Confidence Interval.

Supplementary table 3. Crude within-individual associations of sociodemographic factors, lifestyle factors, and work characteristics with sleep quality, sleep duration, and sleep disturbances among 467 hospital workers, estimated with univariate between-within Poisson regression analyses.

	Poor sleep quality	Non-recommended sleep duration	Sleep disturbances
	RR (95% CI)*	RR (95% CI)*	RR (95% CI)*
**Nurse (ref. other occupation)**	1.00 (0.07, 14.70)	0.56 (0.07, 4.48)	1.86 (0.22, 16.08)
**Body Mass Index (ref. normal weight)**			
Overweight	1.78 (0.22, 14.13)	1.44 (0.27, 7.62)	0.70 (0.13, 3.66)
Obese	1.78 (0.15, 21.39)	2.66 (0.36, 19.87)	0.83 (0.11, 6.37)
**More physical activity (in hours/week)**			
Sports	0.96 (0.83, 1.10)	0.91 (0.83, 1.01)	0.99 (0.89, 1.11)
Occupational physical activity	1.01 (0.98, 1.03)	0.99 (0.97, 1.01)	1.00 (0.98, 1.02)
Other physical activity	1.01 (0.99, 1.03)	1.01 (0.99, 1.02)	1.00 (0.98, 1.02)
**Smoking (ref. never smoker)**			
Former smoker	1.94 (0.40, 9.38)	0.75 (0.21, 2.70)	1.48 (0.40, 5.52)
Current smoker	1.56 (0.22, 11.34)	1.05 (0.21, 5.19)	0.78 (0.15, 3.95)
**Glasses of alcohol per week (ref. 0 glasses)**			
1-7 glasses per week	1.32 (0.47, 3.66)	1.19 (0.53, 2.69)	0.91 (0.39, 2.13)
>7 glasses per week	1.32 (0.31, 5.55)	1.33 (0.42, 4.20)	0.91 (0.28, 2.96)
**Daily screen use 1 hour before sleep**	1.32 (0.64, 2.74)	1.16 (0.64, 2.11)	0.87 (0.48, 1.58)
**Working hours (ref. ≤24 hours per week)**			
25-35 hours per week	0.59 (0.16, 2.24)	0.41 (0.13, 1.29)	0.97 (0.33, 2.80)
≥36 hours per week	0.99 (0.19, 5.17)	0.75 (0.19,2.96)	0.94 (0.24, 3.65)
**Years with night work (ref. <10 years)**			
10-19 years	1.31 (0.30, 5.74)	1.22 (0.37, 4.02)	0.71 (0.20, 2.47)
≥20 years	1.74 (0.22, 13.67)	2.40 (0.46, 12.56)	0.59 (0.11, 3.26)
**Night shifts per month (ref. 1-2 per month)**			
3-4 per month	1.41 (0.46, 4.32)	1.91 (0.77, 4.73)	0.64 (0.28, 1.47)
≥5 per month	1.22 (0.34, 4.31)	1.52 (0.55, 4.21)	0.51 (0.19, 1.35)

Numbers depicted in bold are statistically significant.

Sleep quality: 0 = good, 1 = bad. Sleep duration: 0 = recommended sleep duration (7-9 hours per day), 1 = non-recommended sleep duration (<7 of ≥ 9 hours per day). Sleep disturbances: 0 = less disturbances (lowest 75% sleep disturbances), 1 = more disturbances (highest 25% sleep disturbances).

*RR = relative risk, 95% CI = 95% Confidence Interval.
